# Return to Sports and Activities after Arthroscopic Treatments for Rotator Cuff Lesions in Young Patients Less Than 45-Years-Old: A Systematic Review

**DOI:** 10.3390/jcm13133703

**Published:** 2024-06-25

**Authors:** Mohamad K. Moussa, Elena Lang, Antoine Gerometta, Karam Karam, Mickael Chelli, Olivier Grimaud, Nicolas Lefèvre, Ryan Bou Raad, Yoann Bohu, Frédéric Khiami, Zeinab Khalaf, Pierre Abadie, Alexandre Hardy

**Affiliations:** 1 Groupe Hospitalier Sélestat-Obernai, 67600 Sélestat, France; 2 Clinique du Sport, 75005 Paris, France; elena.lang.okb@gmail.com (E.L.); antoine.gerometta@gmail.com (A.G.); karamarkaram@gmail.com (K.K.); docteurlefevre@gmail.com (N.L.); ryan.bouraad@gmail.com (R.B.R.); zaynab.khalaf90@gmail.com (Z.K.); dr.hardy@chirurgiedusport.com (A.H.); 3 Institut de Chirurgie Réparatrice, 06000 Nice, France; mikael@easymedstat.com; 4 Centre de Chirurgie Orthopédique et Sportive (CCOS), 33700 Mérignac, France; pierreabadie1@gmail.com

**Keywords:** rotator cuff tear, arthroscopic repair, shoulder arthroscopy, return to sport, return to work

## Abstract

**Background:** To evaluate the outcomes of arthroscopic treatment of rotator cuff tear (RCT) in individuals under 45 years, focusing on their ability to return to sports (RTS) and work, along with different patient-reported outcomes (PROMs). **Methods:** Adhering to the Preferred Reporting Items for Systematic Reviews and Meta-Analyses (PRISMA) 2020 guidelines, this systematic review encompassed articles that studied the outcomes of arthroscopic treatment of RCT in the young population (those under 45 years old). The literature search was conducted in PubMed/Medline and EMBASE until 21 May 2024. The primary outcome was the RTS, with secondary outcomes including the return to work and various PROMs. These PROMs included the American Shoulder and Elbow Surgeons (ASES) score and 10 other PROMs. **Results:** Out of 6267 articles, 15 met the inclusion criteria, involving 659 patients, predominantly male athletes with a weighted mean age of 28.3 years. The RCT etiology (14 studies) was primarily traumatic (72.3%), followed by chronic microtrauma in overhead athletes (16.8%) and non-traumatic (10.9%). The RTS rate (12 studies) varied between 47% and 100%, with a cumulative rate of 75.2%. The cumulative rate of return to the same or higher RTS level (11 studies) was 56.1%. Excluding non-athletes and patients treated with debridement, the RTS rates increased to 79.8% (143/179) overall, with a 61% (108/177) rate of returning to the same or higher level. The return to work (3 studies) was successful in 90.6% of cases. Postoperative ASES scores (5 studies) improved markedly to a weighted post-operative mean of 75.6, with similar positive trends across other PROMs. **Conclusions:** Young adults undergoing arthroscopic RCT repair typically experience a 75% RTS rate at any level, and 56.1% RTS at the same level. Excluding non-athletes and debridement patients, RTS rates rise to 79.8% (143/179), with 61% (108/177) achieving the same or higher level. Level of evidence: IV, systematic review including case series.

## 1. Introduction

Rotator cuff tear (RCT) is a well-known pathology in the general population and one of the leading causes of shoulder pain complaints [1]. The incidence of the disease is lower in young individuals. RCT is more symptomatic before the age of 40, unlike the same pathology in older subjects [2]. This is due to a different pathophysiology, with lesions in older subjects often being related to progressive degeneration of tissue quality. In contrast, tears in young subjects are more often traumatic in origin, whether they are single or repeated traumas [3].

Several studies have shown better healing abilities of RCTs in young patients [4,5]. Boileau et al. found 43% healing after arthroscopic repair of an isolated supraspinatus tear in those over 65, compared to 95% in those under 55 [4].

Studies focusing on young subjects remain rare, yet understanding the functional prognosis of these lesions is essential in this population [6,7]. The only systematic review on RCTs in young subjects was conducted in 2015 by Lazarides et al., identifying 12 articles published between 1985 and 2011 [6]. While that study asserted that RCT in younger patients presents a distinct pathology compared to those in older individuals, the inclusion of heterogeneous studies, with as many as 50% involving open surgery, limits its relevance. It seems important to update this systematic literature review, while focusing on arthroscopic procedures, given the evolution of surgical practices concerning RTC repairs and perioperative care. Indeed, studies have shown that the rate of complications, whether surgical or medical, was lower with arthroscopic repair than with open repair [8].

The primary objective of this systematic review was to assess the return to sport (RTS) in individuals younger than 45 years who underwent arthroscopic RCT treatment. The secondary aim was to evaluate the overall impact of RCT treatment on their functional status as measured by their return to work, as well as by different patient-reported outcome measures (PROMs) across the included studies.

The primary hypothesis posited that this population would exhibit high RTS rates at the same pre-injury level, along with distinct profiles in patient-reported outcome measures.

## 2. Material and Methods

This systematic review (SR) was conducted following the 2020 PRISMA (Preferred Reporting Items for Systematic Reviews and Meta-Analyses) guidelines [9].

### 2.1. Eligibility Criteria

The inclusion criteria: an article studying the outcomes of arthroscopic repair of RCT in a young population (<45 years old), regardless of whether they are full-thickness or partial-thickness tears on the articular or acromial side of the cuff. If a study with a wider range age group included a subgroup analysis for the target age (<45 years old) it was included.

The exclusion criteria were open repairs, fundamental studies, case reports, concomitant treatment of RCT associated with shoulder dislocation, and studies with a mean age of patients over 45 years old except when these studies performed a subgroup analysis for patients under 45 years old.

### 2.2. Outcome Measures

The primary outcome of this systematic review was the RTS. This included the evaluation of the rate, quality, and time to RTS. A subgroup analysis was performed to analyze these rates in athletes who were treated with RCT repair rather than debridement.

The secondary outcomes were the functional status as measured by their return to work, as well as by different patient-reported outcome measures (PROMs) across the included studies. PROMs included: American Shoulder and Elbow Surgeons (ASES) score; Single Assessment Numeric Evaluation (SANE) score; Visual Analog Scale (VAS) for pain; Constant–Murley Shoulder Outcome Score (Constant score); Simple Shoulder Test (SST); Western Ontario Rotator Cuff Index (WORC); Oxford Shoulder Score (OSS); University of California at Los Angeles Shoulder Rating Scale (UCLA); Disabilities of the Arm, Shoulder, and Hand (DASH) score and its sports-specific module (QuickDASH); Athletic Shoulder Outcome Rating Scale (ASORS); and the 12-Item Short Form Health Survey (SF-12), including Physical (PSF) and Mental (MSF) Component Summaries.

It also involved examining the specific characteristics of this young population experiencing RCT.

### 2.3. Search Strategy

A comprehensive literature search was conducted in the PubMed/Medline Ovid database until 21 May 2024. The search strategy/strings were registered in PROSPERO (Registration number: CRD42023439293). It involved a combination of free text words and MeSH (Medical Subject Headings) terms to devise a series of search strings. These strings incorporated a range of keywords and phrases including: “rotator cuff tear,” “young patients,” “younger,” “45 years old,” “return to sport,” “return to work,” “tear of the rotator cuff,” “traumatic rotator cuff rupture,” “traumatic rotator cuff tear,” “traumatic rotator cuff lesion,” “rotator cuff rupture,” “traumatic”, “functional outcomes,” “patient-reported outcomes,” “clinical outcomes,” “functional scores,” and “muscle strength”. These terms were used both individually and in various combinations separated by “AND” or “OR” to expand the scope of our search.

### 2.4. Study Selection

Two independent reviewers performed the search (MM, and EL) using the Rayan^®^ Software (version 2024) [10]. A three-step process was followed. The first step involved title screening for inclusion based on eligibility criteria and duplicate exclusion. Duplicates were removed by an automatic tool. The second step consisted of abstract analysis to confirm inclusion. Finally, the third step entailed full-text manuscript analysis for data extraction. In cases of disagreement, a mutual agreement was reached between the two authors on whether to include it in the study or not.

### 2.5. Data Extraction

The data from the included studies were organized for further analysis. The relevant data for each study included the number of patients, follow-up duration, patient demographics (age and sex), activity level, etiology of the tear, type of RCT, involved tendons, study type, patient treatment, surgical techniques used, associated lesions, primary outcomes, and observations. Whenever possible, data from heterogeneous populations or involving distinct treatment modalities were separated into subgroups for analysis.

### 2.6. Evaluation of the Quality of the Included Studies

The methodological quality of the studies included in our review was evaluated using the Methodological Index for Nonrandomized Studies (MINORS) tool [11]. This checklist, designed specifically to assess nonrandomized studies, comprises 12 elements that examine the study’s objectives, inclusion and exclusion criteria, data collection and analysis methods, and result reporting.

The evaluation of studies was performed independently by two authors. Any disagreements in the scoring were resolved through discussion until a consensus was reached.

### 2.7. Data Synthesis

In the review, a narrative synthesis was performed. Data were synthesized from studies that provided sufficient quantitative and qualitative information related to the primary and secondary outcome measures. A threshold of a minimum of five studies was required for a specific outcome to be considered for synthesis.

Given the nature of the analysis, a meta-analysis or quantitative synthesis was not conducted. Significant heterogeneity was observed among the selected studies, so the results are presented as weighted means, minimum–maximum ranges, and percentages. No homogeneity test was used.

## 3. Results

### 3.1. Included Studies

**Figure 1:**
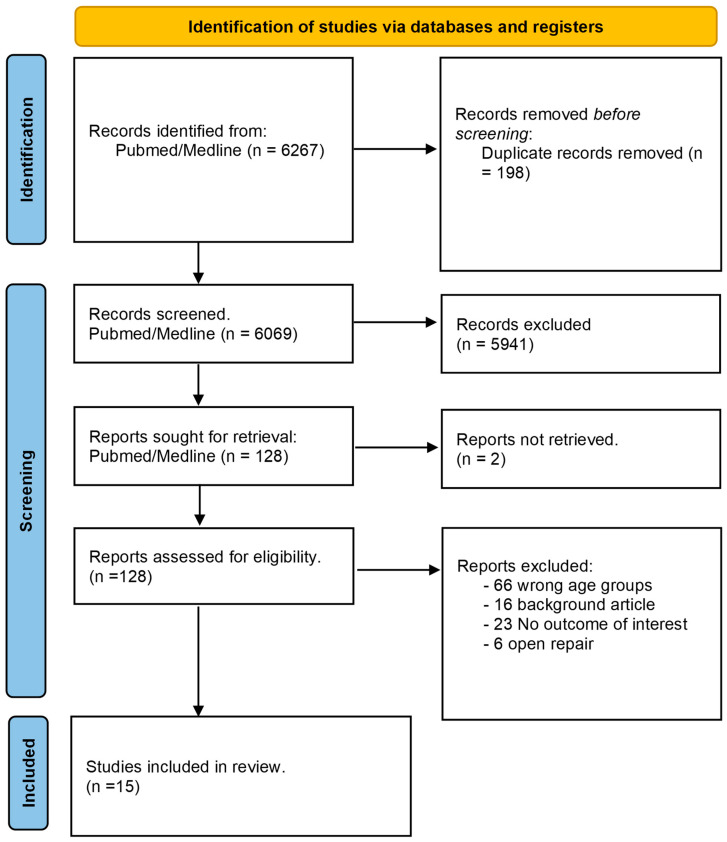
Prisma 2020 flow diagram for the systematic review.

A total of 6267 articles were initially identified through database searches. After removing duplicates (178), 6049 articles remained for title and abstract screening. Following the screening process, 122 articles were considered potentially eligible for inclusion, and their full-text versions were assessed for eligibility. Ultimately, 15 articles (Table 1, Figure 1) were included in the SR, encompassing a total of 659 patients.

### 3.2. Study Characteristics and Quality (Table 1)

Methodological quality, as assessed by the MINORS scores, varied with scores ranging from 6 to 16. The Level of Evidence (LOE) ranged from three (in three studies) to four (in 12 studies).

### 3.3. Patient Characteristics (Table 1)

There was a clear male dominance, with 525 (79.7%) male and 134 (20.3%) female participants reported across all studies.

The patient age spanned from adolescents to middle-aged adults, with a weighted mean of 28.3 years across studies. The population profile was predominantly composed of individuals engaged in sports (457 out of 659, 69.4%), which was further broken down into elite/professional athletes (136 out of 457, 29.7%) and other sports participants (321 out of 457, 70.3%). In addition to the sports participants, there were military personnel (79 out of 659, 12%) and manual workers (43 out of 659, 6.5%). The specific profile was not reported for 80 patients (12.1%).

The etiology of RCT in patients <45 years was reported in all studies except one [20], leaving a total of 589 patients for etiology analysis. The RCT etiology was primarily traumatic, constituting 72.3% with 426 out of 589 cases. Overhead sports activities were implicated as a chronic microtraumatic cause in 16.8% of cases, totaling 99 instances. Non-traumatic origins were identified in 64 cases, representing 10.9%.

The supraspinatus (SSP) tendon was most frequently involved, either alone or with other tendons. Both full and partial thickness tears were commonly reported.

The surgical techniques used involved single or double-row suture anchor techniques (Krishnan 2008 [12]; Lin 2013 [7]). Additional procedures frequently performed included debridement, tenodesis, and acromioplasties to address concurrent pathologies (Krishnan 2008 [12]; Parnes 2018 [19]).

The mean follow-up ranged from 16.9 to 104.51 months across the studies (weighted mean = 46.2).

### 3.4. Return to Sport (Table 2)

The rate of RTS varied, as noted in 12 studies encompassing 449 patients, with rates ranging from 47% to 100%, with a cumulative rate 75.2% (338/449). The quality of RTS was reported in 11 studies including 410 patients. The rate of RTS to the same or higher level ranged from 35.2% to 100% with a cumulative rate of 56.1% (230/410). Time to RTS was quantified in 6 studies covering 196 patients, with reported timeframes ranging from 3 to 11 months postoperatively and a weighted mean of 5.2 months.

When isolating studies evaluating purely athletes’ patients, 10 studies were found. The rate of RTS was 238/347 (69.5%). The rate of return to the same or higher level was 209/387 (54%).

When further excluding patients who were treated with debridement, only eight studies were left. The rate of RTS was 143/179 (79.8%). The rate of return to the same or higher level was 108/177 (61%).

**Table 2:** Summary of studies reporting the RTS outcomes post-arthroscopic rotator cuff tear repair in individuals under 45 years.

Study	N	Loss to Follow-Up or Excluded	Type of Sport	Level of Sport	Rate of Return to Sport	Rate of Return to Same or Higher Level	Time to Return to Unrestricted Sport (months)	Time of Authorization of Return to Unrestricted Work as per Study Protocol
2008, Krishnan et al. [12]	23	0	Worker compensation: 20. Professional athletes: 3	Professional vs recreational	23/23 (100%)	21/23 (90%)	NR	Resistance exercises began at week 10. Activity progression was allowed as tolerated following this and full release to all activity and work (including sports) was allowed 6 months after surgery.
2008, Reynolds et al. [13]	82	15	Professional elite overhead throwing athletes (Baseball pitchers)	Professional	51/67 (76.1%)	37/67 (55.2%)	5.6 (SD = 2.6)	Early pain and inflammatory control phase, protection phase, gradual return of range of motion, strengthening, and gradual return to competition.
2009, Tambe et al. [14]	11	0	Professional elite rugby players	Professional	10/11 (90.9%)	10/11 (90.9%)	5.75 (SD = 2.42, range 3–11)	Accelerated rehabilitation program. Depending on the athletes’ progress, resistance exercises are introduced after approximately six weeks, along with skills training. Simulated tackling is started at about two to three months. Impact and tackle bag training is not started until the athletes have achieved satisfactory movement, strength, isokinetic and proprioceptive criteria.
2012, Van Kleumen et al. [15]	17	0	Baseball, overhead-throwing sports	Interscholastic, Intercollegiate	8/17 (47%)	6/17 (35.2%)	NR	NR
2013, Eisner et al. [16]	30	17	Adolescent overhead athletes	NR	9/13 (69.2%)	9/13 (69.2%)	NR	6-week course of physical therapy, which consisted of rotator cuff stretching and strengthening, and scapular stabilizing exercises under the direction of licensed physical therapists before being offered surgical treatment.
2018, Azzam et al. [17]	32	5	Athletes: Football, baseball, basketball, softball, wrestling, motocross, track, volleyball, bull riding, cross country, mixed martial arts	Athletes, NR otherwise	25/27 (93%)	17/27 (62.9%)	NR	Progression to sport-specific exercises began at 5 to 6 months, once the patient had obtained full motion and full strength. The patient was released to full activities when he or she had completed a sport-specific rehabilitation progression or interval training program, depending on the sport involved.
2018, Merola et al. [18]	38	0	Professional overhead athletes.	Professional	33/38 (87%)	19/38 (50%)	6 (SD = 0.7) months	Wearing a sling for the first 3 weeks, passive mobilization and active-assisted exercises in a pool in the pain-free range of motion (ROM) up to the 6th week, and thereafter active exercises with and without resistance.
2018, Parness et al. [19]	42	0	Military	Recreational and military job athletes	40/42 (95.2%)	40/42 (95.2%)	6 (SD = 0.7) months.	Return to full military job activity and contact sports was allowed 6 months after surgery.
2018, Rossi et al. [20]	70 *	0	NR for subgroup analysis	NR for subgroup analysis	NR for subgroup analysis	NR for subgroup analysis	5.2 (SD = 2) months	Return to competition was allowed when the patient was pain free, full-shoulder ROM had been achieved, and shoulder strength was near the same as before the injury
2019, Erickson et al. [21]—Debridement	130	0	Baseball (Major league)	Professional	Debridement: 75/130 (57.6%)	Debridement: 55/130 (42.3%),	NR	NR
2019, Erickson et al. [21]—Repair (same study	21	0	Baseball (Major league)	Professional	Repair: 7/21 (33.3%)	Repair: 3/19 (14.3%)	NR	NR
2021, Castagna et al. [23]	3	0	Professional soccer goalkeepers	Professional	3/3 (100%)	3/3 (100%) (Objective metrics: 96.7% ± 5.8)	4.76 (SD = 0.97 months. (reported as 20.7 ± 4.2 weeks)	NR
2021, Davey et al. [24]	20	0	2 professional athletes(10.0%) 16 competitive athletes (80.0%) 2 recreational athletes (10.0%); there were 15 collision athletes (75.0%)	Professional athlete 10%. Competitive athlete 80%. Recreational athlete 10%	17/20 (85%) for all 16/18 (88.9%) for professional and competitive	10/20 (50%)	5.8 (SD = 2.8)	A controlled return to contact in training was allowed after 12 weeks if comfortable, whereas a return to full contact and competition usually followed within the next 3 months.
2022, Scanaliato et al. [25]	42	5	Military	military patients	37/37 (100%)	NR	NR	Return to unrestricted active duty and contact sports were permitted 6 months postoperatively.

* This study underwent subgroup analysis of younger patients group without precising the number of patient of patients. N correspond the number of patients in the whole population.

### 3.5. Return to Work (Table 3)

Four studies reported on the return to work corresponding to a pooled population of 160 patients. A cumulative rate of 90.6% (145/160) successfully returned to work post-intervention. Krishnan et al. [12] reported a return rate of 91.3% (21/23) among patients involving worker compensation and professional athletes. Similarly, Lin et al. [7] documented an 88.6% (47/53) return rate for work-related injuries. Military personnel in the studies by Parness et al. [19] and Scanaliato et al. [25] exhibited return rates of 95.2% (40/42) and 88.1% (37/42), respectively. There are limited reports on the time to return to work. However, across the included studies, the consistent time for authorization of return to unrestricted work was at the 6-month mark post-procedure.

**Table 3:** Summary of studies reporting the return-to-work outcomes post-arthroscopic rotator cuff tear repair in individuals under 45 years.

Study	N	Type of Work	Rate Of Return To Work	Time to Return to Work	Time of Authorization of Return to Unrestricted Work as by Study Protocol	Follow-Up (months)
2008, Krishnan et al. [12]	23	Worker compensation: 20. Professional athletes: 3	21/23 (91.3%)	NR	6 months	26 (SD = NR, 24 to 29)
2013, Lin et al. [7]	53	Work related: 23	47/53 (88.6%)	NR	6 months	37.8 (SD = 10.2, range 13.8 to 59.1)
2018, Parness et al. [19]	42	Military	40/42 (95.2%)	NR	6 months	41 (SD = NR, 24 to 66)
2022, Scanaliato et al. [25]	42	Military	37/42 (88.1%)	NR	6 months	104.51 (SD = 7.67, NR)
Total	160	Military: 84 Worker compensation: 20 Athletes: 3	145/160 (90.6%)	NR	6 months	

### 3.6. Functional Scores

The ASES score was the most commonly applied measure, reported in seven studies [12,17,20,22,24,25]. Pain assessment was predominantly performed using the VAS score in five studies [19,20,22,24,25]. For functional outcomes, the Constant score [14] and the UCLA score [22] were each employed in one study. The SANE score was utilized in three studies [7,16,25], while the KJOC shoulder and elbow scores [15,18] were reported in two studies.

Other PROM included the Oxford score [14], the SST score [7], the SF-12 thought PSF and MSF scores [13], and the WORC index [17], which were each also used in one study.

### 3.7. ASES Score (Table 4)

Preoperative ASES scores were reported in five studies [12,19,20,22,25], comprising a collective sample size of 253 patients. Scores ranged from 23.4 to 43.3 (weighted mean = 42.4).

Postoperative ASES scores were documented in eight studies, encompassing 428 patients in total [7,12,17,19,20,22,24,25]. Following surgery, patients exhibited a high ASES across the board, with means ranging from 72.2 to 93 (weighted mean = 75.6).

**Table 4:** Summary of studies reporting the ASES outcomes post-arthroscopic rotator cuff tear repair in individuals under 45 years.

Study	N	Pre Operative ASES	Post Operative ASES	*p*-Value
2008, Krishnan et al. [12]	23	42 (SD = NR, range 22 to 60).	92 (SD = NR, range, 65 to 100)	<0.01
2013, Lin et al. [7]	53	NR	84.6 (SD = 16.8, range, 21.6 to 100.0)	N/A
2018, Azzam et al. [17]	32	NR	93 (SD= 16.8, range, 21.6 to 100.0)	N/A
2018, Parness et al. [19]	42	38.97 (SD = 12.70)	89.8 (SD = 14.26)	<0.01
2018, Rossi et al. [20]	Subgroup analysis n not available (N = 70 *)	43.3 (SD = 1)	88.4 (SD = 2)	NR
2020, Kaptan et al. [22]	151	Partial + Full thickness: 23.8, (SD = NR, range, 8.3 to 36.6), Full thickness: 23.4 (SD = NR, range, 8.3 to 36.6)	Partial + Full thickness: 72.2 (SD = NR, range, 11.6 to 88.3) Full thickness: 72.3 (SD = NR, range, 11.6 to 88.3)	<0.01
2021, Davey et al. [24]	20	NR	92.4 (SD = 4.6, range 83.3–100)	N/A
2022, Scanaliato et al. [25]	37	41.00 (SD = NR, range, 34.74 to 47.26)	Midterm: 90.84 (SD = NR, range, 85.78 to 95.90) Final follow up: 88.68 (SD = NR, range 82.69 to 94.66)	<0.001
* Subgroup analysis available				

* This study underwent subgroup analysis of younger patients group without precising the number of patient. N correspond the number of patients in the whole population.

### 3.8. SANE Score (Table 5)

Preoperative SANE score was reported in one study by Scanaliato et al. reporting a mean of 48.24 (SD = NR, range, 39.52 to 56.96) [25].

Postoperative SANE scores were documented in three studies, encompassing 120 patients in total. Following surgery, high SANE scores were recorded with means ranging from 80.6 to 89.19 (weighted mean = 83.7) [7,16,25].

**Table 5:** Summary of studies reporting the SANE score outcomes post-arthroscopic rotator cuff tear repair in individuals under 45 years.

SANE Score	N	Preoperative	Post Operative	*p*-Value
2013, Eisner et al. [16]	30	NR	80.6 (SD = 17.1)	N/A
2013, Lin et al. [7]	53	NR	80.8 (SD = 20, range, 10 to 100)	N/A
2022, Scanaliato et al. [25]	37	48.24 (SD = NR, range, 39.52 to 56.96)	Mid-term: 89.19 (SD = NR, range,82.81 to 95.57) Final follow up: 87.32 (SD = NR, range, 80.72 to 93.93)	<0.001

### 3.9. VAS Score (Table 6)

Preoperative VAS scores were reported in three studies, comprising a collective sample size of 129 patients [7,22,25]. Scores ranged from 7.9 to 8.09 for partial and full thickness conditions (weighted mean =8.2). Postoperative VAS scores were documented in six studies, encompassing 272 patients in total. Following surgery, patients had low VAS scores across the studies, with means ranging from 0.7 to 3.1 (weighted mean= 1.3).

The reduction in VAS score (calculated from the two studies reporting preoperative data) was significant, with *p*-values ranging from <0.01 to <0.0001.

**Table 6:** Summary of studies reporting the VAS outcomes post-arthroscopic rotator cuff tear repair in individuals under 45 years.

VAS	N	Preoperative	Post Operative (Final Follow-Up)	*p*-Value
2013, Lin et al. [7]	53	NR	1.2 (SD = 2, range 0 to 10)	N/A
2018, Parness et al. [19]	42	8.09 (SD = 1.51)	1.19 (SD = 1.85)	<0.01
2018, Rossi et al. [20]	70 *	NR	1.1 (SD = 2)	N/A
2020, Kaptan et al. [22]	50	Partial + full thickness: 7.9 (SD = NR, range 5 to 10) Full thickness: 8.4 (SD = NR, range 6 to 10)	Partial + full thickness: 3.1 (SD = NR, range 1 to 10) Full thickness: 2.3 (SD = NR, range 1 to 4)	<0.01 <0.01
2021, Davey et al. [24]	20	NR	0.7 (SD = NR, range 0 to 3)	N/A
2022, Scanaliato et al. [25]	37	8.03 (SD = NR, range 7.29 to 8.77)	Mid term: 1.14 (SD = NR, range, 0.43 to 1.85) Final follow up: 1.16 (SD = NR, range, 0.44 to 1.88)	<0.0001 <0.0001

* This study underwent subgroup analysis of younger patients group without precising the number of patient. N correspond the number of patients in the whole population.

### 3.10. Constant Score

The Constant score was recorded in two studies. Tambe et al. showed a remarkable increase from a mean pre-operative score of 44 to 95 at the 3-month follow-up, further progressing to an almost perfect score of 99 at the final follow-up [14]. On the other hand, Lin’s 2013 study, which does not provide a pre-operative score, reported a high post-operative Constant score of 81.7 [7].

### 3.11. SSV Score

Parness et al. (2018) documented an increase from a preoperative score of 47.88 (SD = 19.56) to a postoperative score of 89.45 (SD = 14.04) (*p* < 0.01) [19]. Davey (2021) reported a high postoperative SSV of 87.0 (SD = 10.2), although the preoperative score was not provided [24].

### 3.12. KJOC Shoulder and Elbow Score

Merola et al. (2018) reported an increase from a preoperative score of 55.6 (SD = 1.2) to a postoperative score of 73.1 (SD = 7.8), with the difference being statistically significant (*p* < 0.0001) [18]. Van Kleunen et al. (2012) did not report a preoperative score but noted postoperative scores of 66 for full-thickness injuries and 55 for partial-thickness injuries [15].

### 3.13. UCLA Score

In the study by Kaptan et al., patients with RCT had an average preoperative UCLA score of 12.7, with a range of 9 to 22, and the subgroup with full thickness tears had a slightly higher average of 12.9, with the same range [22]. Following surgery, the scores increased notably to 26.1, ranging from 8 to 35 for the combined group (*p* < 0.01), and to 26.5, ranging from 8 to 35 for the full thickness group (*p* < 0.01), demonstrating a significant improvement in shoulder function.

### 3.14. WORC Score

The WORC score was used by Azzam et al. who found an average postoperative score of 89% (range 60–100%, SD = 13%) among patients who underwent rotator cuff repair, indicating a generally high level of function following surgery [17].

### 3.15. Oxford Score

Tambe et al. reported that the Oxford Shoulder Score improved from 34 preoperatively to 12 postoperatively for elite rugby players who underwent arthroscopic rotator cuff repair.

### 3.16. SST Score

The SST score was used by Lin et al., who reported a mean postoperative SST score of 10.7 (range, 3.0 to 12.0; SD, 2.3), indicating a favorable outcome following arthroscopic rotator cuff repair.

### 3.17. QuickDash, Quickdash Sport

Eisner et al. found that following surgery, adolescent patients with RCTs had average QuickDASH and QuickDASH Sports module scores of 8.1 and 19.5, respectively, indicating a moderate level of disability or difficulty in performing specific activities. They suggest that while improvements were noted post-surgery, adolescents may still experience certain limitations in shoulder function, particularly during sports activities, which could impact their overall athletic performance and recovery.

### 3.18. ASORS, SF-12, PSF, MSF

In Reynolds et al.’s study on pitchers, the best Athletic Shoulder Outcome Rating Scale results post-surgery indicated 52.9% excellent, 23.5% good, 11.7% fair, and 11.7% poor. When considering their current status at the time of follow-up, the ratings shifted to 39.4% excellent, 18.2% good, 24.2% fair, and 18.2% poor. The SF-12 scores were reported as being above average, with a Physical Component Summary (PSF-12) mean of 55.04 (SD = 4.48) and a Mental Component Summary (MSF-12) mean of 56.49 (SD = 4.11).

## 4. Discussion

The main finding of this systematic review is that young patients (under 45 years old) undergoing arthroscopic RCT repair generally exhibit high rates of RTS at any level, around 75.2%, yet demonstrate a lower-than-expected rate, 56.1%, of RTS at the same level compared to older age groups documented in the literature. Excluding non-athletes and patients treated with debridement, the RTS rates increased to 79.8% (143/179) overall, with a 61% (108/177) rate of returning to the same or higher level. These findings provide athletes with a reasonable expectation as to whether they will be able to return to their sport (1 out of 4 will not).

One possible explanation for the lower-than-expected outcomes in younger patients is that they often have higher physical demands and greater expectations for their performance levels compared to older patients. Younger athletes may also engage in more strenuous activities, placing greater stress on the repaired tendon, which could impact the overall success and satisfaction with their return to sport. Additionally, the nature of their athletic activities may require a higher level of function and strength, making it more challenging to return to pre-injury performance levels. The observation that athletes have higher RTS rates than amateurs, despite their higher initial performance levels, can be justified by the fact that athletes often have access to intensive, sport-specific rehabilitation programs, enhancing their recovery and RTS at the same level.

For example, Krishnan et al. began resistance exercises at week 10 and allowed full activity and work, including sports, by six months post-surgery [12]. This protocol contributed to their high RTS rates, with 100% (23/23) returning to sports and 90% (21/23) returning to the same level. Reynolds et al. detailed a phased approach, starting with pain and inflammatory control, followed by protection, gradual return of range of motion, strengthening, and ultimately a return to competition [13]. This approach resulted in a 76.1% RTS rate (51/67) among professional elite overhead throwing athletes, with 55.2% (37/67) returning to the same level. Tambe et al. implemented an accelerated rehabilitation program, introducing resistance exercises at six weeks, skills training at two to three months, and full contact training only after achieving satisfactory movement, strength, isokinetic, and proprioceptive criteria. This resulted in a 90.9% RTS rate (10/11) at the same level [14].

## 5. RTS

Our study demonstrated RTS rates ranging from 47% in college athletes [15] to 100% in professional athletes with structured rehabilitation [12], with a cumulative rate of 75.2% in the studied young population. These data present a notable similarity to the findings of Migliorini et al., who reported a 75.4% RTS rate at a mean of 6.4 months post-arthroscopic rotator cuff repair [26]. The population in their review is slightly older, with a mean age of 37.2 years, and it includes several studies where the mean patient age exceeds 50 years, reflecting a high potential for RTS regardless of age following arthroscopic RCT repair [26]. Similarly, the rate of RTS in Klouche et al.’s systematic review was 84.7% [27]. Their study included 635 (93%) athletes with a mean age of 42.6 years, ranging from 15 to 81 years, indicating a broad age spectrum [27]. In contrast, Giberson-Chen et al. reviewed 26 studies with 1228 baseball players post-shoulder surgery, noting a lower RTS rate after RCT surgery at 53.5% [28].

A special consideration in our study pertains to military personnel, as evidenced by Parness’ 2018 and Scanaliato’s 2022 studies, which reported high RTS rates of 95.2% and 88.1%, respectively [19,25]. Our study demonstrates a variable rate of RTS at the same level, ranging from 35.2% to 100% with a cumulative rate of 56.1% (230/410). This is also slightly lower than what is reported in the literature for patients with a wider age range. For instance, Klouche et al. found 65.9% of their patients managing to RTS at an equivalent level of play after 4 to 17 months of recovery [27]. However, this percentage declined in patients at a high level of competition. Interestingly, their findings highlight that while the majority of recreational athletes return to their previous level, professional athletes face a more challenging RTS, with only half (49.9%) returning to their pre-injury level [27]. Similarly, baseball players, as described in the systematic review by Giberson-Chen et al., found a RTS to the same level of 27.9% over a follow-up period of 12 to 109.2 months [28]. These figures underscore the variability of RTS outcomes based on the level of sport participation, type of sport, and age demographics. Noteworthily, the definition of same level RTS differs significantly across the studies, with most of them focusing on the patient’s perception of return quality, rather than objective RTS metrics, which were used in only two studies [21,23].

In contrast, open surgical techniques have shown varied results. For instance, open techniques, like those described by Hawkins et al., often involved extensive procedures such as open repairs in the beach chair position with additional acromioplasties, which, while improving pain and function, did not consistently result in high RTS rates [29]. The study by Mazoue and Andrews indicated that a mini-open approach still posed difficulties for professional athletes in returning to competitive levels, with only four out of nine position players returning to the same level or higher [30].

## 6. Return to Work

Although this review could not identify any studies detailing precise return-to-work metrics like sick leave duration or the timeline for transitioning to modified work duties or workplace accommodations, it did report a high rate of return to work, with approximately 90.6% (145/160) successfully resuming their jobs post-intervention. Notably, all studies concurred on a standardized 6-month duration before permitting an unrestricted return to work following surgery. However, the representativeness of the population in these studies is debatable, given that military personnel made up a significant portion (84/160), alongside workers’ compensation cases (20/160), and a minimal number of athletes (3/160). A noteworthy study to compare with these results is the systematic review by Kholinne et al., who reported on return to work with unrestricted age selection, with several included studies having lower means among those over 45 years old [31]. Their systematic review reported an overall return to work rate of 83.8% with a mean time of 6.29 months to return to work, indicating a substantial proportion of individuals could resume their employment within a similar timeframe as our findings, albeit with a slightly lower return rate. The higher rate of return-to-work rates in our review (90.6%) than that of Kholinne et al. (83.8%) could suggest that age and other demographic factors may influence the likelihood of returning to work post-surgery [31].

## 7. Functional Scores

Our study demonstrated that the collated data across various scoring systems, including ASES, SANE, VAS, UCLA, Constant, SSV, KJOC, Oxford, SST, and QuickDASH scores, provide a comprehensive insight into the efficacy of shoulder arthroscopy in the studied young population. Our findings are consistent with those of MacKechnie et al., who investigated full-thickness rotator cuff repairs in patients under 55 years and observed an 82% satisfaction rate postoperatively [32]. In addition to an average postoperative ASES score of 82.0, they noted a weighted mean Constant score of 72.7, and SANE score of 82.1, suggesting robust patient-reported outcomes [32]. In Miglorini et al., all of the PROMs were considered improved from baseline to last follow-up in overhead athletes [26]. All of the PROMs improved at last follow-up: KJOC (MD + 25.0; *p* = 0.02), VAS (MD − 5.0; *p* = 0.003), Constant (MD + 40.5; *p* < 0.0001), UCLA-S (MD + 31.2; *p* = 0.006), and ASES (MD + 40.0; *p* < 0.0001).

On the other hand, the functional outcomes in our review are echoed in the literature focusing on older patients. For instance, Altintas et al. conducted a systematic review, uncovering substantial improvements in clinical and functional outcomes post-RCT repair in patients aged ≥ 70 [33]. Analyzing eleven studies with 680 patients, they reported ASES score enhancements from a preoperative 44.2 to a postoperative 87.9, and Constant scores rising from 41.7 to 70.8. Despite a retear rate of 27.1% among the 513 shoulders assessed postoperatively, their work underlines RCT repair as a viable joint-preserving treatment, offering substantial pain relief (VAS scores dropping from 5.5 to 1.3) and overall satisfaction for elderly patients [33]. Similarly, Hsieh et al.’s systematic review and meta-analysis discerned no significant disparities in surgical outcomes between older (over 65 or 70 years) and younger patients [34]. Their analysis included 671 participants and reported no marked differences in Constant score improvements, re-tear rates (OR: 0.98; 95% CI: 0.47 to 2.02), or shoulder function and pain levels (VAS pain reduction MD: −0.33; 95% CI: −0.80 to 0.15) [34].

## 8. Limitation

The limitations of this study primarily stem from the reliance on studies with low levels of evidence, predominantly level III and IV, which inherently restricts the strength of its conclusions. The quality of these studies is further impacted by several methodological weaknesses noted in the MINORS evaluation. One limitation of this study is that the varying physical demands, injury mechanisms, and rehabilitation protocols specific to different sports were not individually accounted for. Additionally, the included studies exhibit significant heterogeneity in terms of patient demographics, treatment methods, and outcome measures.

Furthermore, the representativeness of the outcomes is constrained by the specific populations studied; most of the research focuses on athletes for the RTS outcome and military personnel for the return-to-work outcome, which may not be reflective of the broader young population experiencing rotator cuff tears.

## 9. Conclusions

Young adults undergoing arthroscopic RCT repair typically experience high RTS rates at any level, with a cumulative rate of 75.2%, but not as high as expected when it comes to returning to the same level of sport, with a cumulative rate of 56.1%. Excluding non-athletes and patients treated with debridement, the RTS rate increases to 79.8% (143/179) overall, with a 61% (108/177) rate of returning to the same or higher level. These findings provide athletes with a reasonable expectation as to whether they will be able to return to their sport. Arthroscopic RCT treatments also lead to substantial functional improvements and successful return to work in 90.6% of cases.

## Figures and Tables

**Table 1:** Overview of the included studies, detailing methodological quality, patient demographics, clinical characteristics, and outcomes for arthroscopic RCT repairs in individuals under 45 years.

Study	MINORS	Type of Study	LOE	Age: Mean (SD, Range)	Sex	n	Site (R/L or D/ND)	Tendon Involved	Partial versus Complete	Activity (Athletes, Worker)	Etiology	Mean Time before Surgery	Technique	Mean Follow Up (months)
2008, Krishnan et al. [12]	10	Case series	4	37 (NR, 21–39)	M: 15, F: 8	23	NR	SSP: 78%, SSP + ISP: 22%	Full thickness	Worker compensation: 20. Athletes: 3	T: 22 (95%)	6 (3–9) months	Double row repair	26 (SD = NR, 24 to 29)
2008, Reynolds et al. [13]	6	Retrospective study	4	25.6 (5.5, NR)	M: 82	82	55 R, 27 L	SSP + ISP	Partial thickness	Elite overhead throwing athletes (Baseball pitchers)	Multifactorial related to overhead-throwing sports	<6-weeks: 6 >6-weeks: 76	Debridement	38 (SD = NR, 18 to 59)
2009, Tambe et al. [14]	8	Retrospective study	4	25.7 (NR, 19–31)	M: 11	11	NR	SSP: 9 SSP + ISP: 1 SSP + SSC: 1	Full thickness	Elite rugby players	T: 100%	1.15 month	Simple or double row repair	18 (SD = NR, 6 to 31)
2012, Van Kleumen et al. [15]	12	Case series	4	19.16 (NR, 16.08–22.92)	M: 17	17		ISP + SLAP	Full thickness: 6, Partial thickness: 11	Baseball, overhead-throwing sports	Multifactorial related to overhead-throwing sports	NR	Simple row or free PDS suture	37.12 (SD = NR, 24 to 55)
2013, Eisner et al. [16]	15	Retrospective cohort	3	15.5 (1.8, 8–18)	M: 16 F: 14	30	R: 24 L: 6	SSP	Partial thickness	Adolescent athletes	T: 30 (100%)	5.8 month	Debridement or repair	16.9 (SD = NR, 8 to 30)
2013, Lin et al. [7]	7	Case series	4	37.5 (16–45)	M: 40 D: 13	53	D: 53%	All types	Full thickness	Work related: 23 (43%)	T: 32 (60%)	NR	83% double-row suture, 17% single-row)	37.8 (SD = 10.2, 13.8 to 59.1)
2018, Azzam et al. [17]	10	Case series	4	16.1 (1.3, 13.2–17.9)	M: 28 F: 4	32	D: 26 ND: 6	SSP: 21 SS: 6 SSP + ISP: 2 SSP+ SS: 1 ISP: 1 SSP + ISP + TM: 1	Full thickness: 13 Partial thickness: 19	Athletes: Football, baseball, basketball, softball, wrestling, motocross, track, volleyball, bull riding, cross country, mixed martial arts	T: 29 (90.6%)	NR	Simple or double row repair	74.4 (SD = 31.2, 24–120)
2018, Merola et al. [18]	12	Retrospective cohort	3	27 (NR, 21–33)	M: 17 F:21	38	R: 30 D: 30	SSP: 28 (74%) SSP+ SLAP: 10 (26%)	Full thickness: 23 Partial: 15	Professional overhead athletes.	Multifactorial related to overhead-throwing sports	10.5 (6–18)	Tear: 1 or 2 triple-loaded, metal suture anchors (Threvo;ConMed, Largo FL, USA) SLAP: double-loaded nonmetallicsuture anchors (Lupine, DePuySynthes; Raynham, MA, USA and Y-knot, ConMed; Largo, FL, USA)	Minimum 24 months
2018, Parness et al. [19]		Retrospective cohort	3	32.7 (NA, 21–39 years)	M: 35 F: 7	42	NR	SSP: 37 SSP + ISP: 2 SSP + ISP + S: 3	Full thickness	Military	T: 24 (68.5%) NT: 18 (31.4%)	23 months	Simple or double row repai	41 (SD = NR, 24 to 66)
2018, Rossi et al. [20]	16	Case series	4	Subgroup analysis: 20–40 years	M: 37, F: 33 *	70 *	D: 43, ND: 27 *	SSP *	Partial thickness (38 bursal, 32 articular) *	Non collision/non overhead = 35. High impact/collision sports = 20. Overhead sport = 15 *	T: 20 NT: 50 *	5.8 *	Simple row repair For <1 cm: 1 anchor used. For >1 cm: 2 anchor used *	54 (SD = NR, 24 to 113) *
2019, Erickson et al. [21]	8	Case series	4	24.6 (4.1)	M: 151	151	D: 141 ND: 10	SSP: 48 SSP + ISP: 68 ISP: 29 SS: 2	Full thickness: 4%, Partial thickness: 96%	Baseball (Major league)	Trauma: 141 (100%)	NR	130 debridement 21 repairs (6 single row, 5 double rows, 10 side to side) Anchors: 2.09 (1.1, range, 1–4)	Minimum 12 months
2020, Kaptan et al. [22]	14	Case series	4	41.4 (3.96, 31–45)	M: 24, F: 26	50	NR	NR	Full thickness: 20, Partial thickness: 30	NR	T: 39 (78%) NT: 11 (22%)	1.38	Double row repair	42.4 (SD = 13.3, 24 to 95)
2021, Castagna et al. [23]	13	Case series	4	28 (6.2, NR)	M: 3	3	D: 1/3, ND: 2/3	NR	NR	Professional soccer goalkeepers	T: 3 (100%)	2.53 (1.84–2.99)	NR	57 (SD = NR, 140 to 130)
2021, Davey et al. [24]	10	Case series	4	25.5 (NR, 18–29)	M: 80%, F: 20%	20	NR	SSP: 18 SS: 2	NR	Collision athlete 75% Overhead sport athlete 20%. Professional athlete 10%. Competitive athlete 80%. Recreational athlete 10%	T: 100%	<3	Double-row repair with medial knots and knotless lateral anchor. Depending on the extent of the tear, 1 or 2 medial-row anchors and 1 or 2 lateral anchors were used	31.8 (SD = 14.7, 15 to 56)
2022, Scanaliato et al. [25]	14	Case series	4	34.03 (NR, 24–39)	M: 33 (89.2%)	37	R: 67.6% (25) D: 75.7% (28)	NR	Full thickness	Military	T: 100%	25.05 ± 28.13 (3–120)	Single-row technique utilized for C1 and C2 tears and a suture bridge double-row repair for C3 and C4 tears.	104.51 (SD = 7.67, NR)

* data not reported for the performed subgroup analysis.

## Abbreviations

RCT	Rotator cuff tear
RTS	Return to sport
PROMs	Patient-reported outcome measures
ASES	American Shoulder and Elbow Surgeons score
SANE	Single Assessment Numeric Evaluation score
VAS	Visual Analog Scale
Constant score	Constant–Murley Shoulder Outcome Score
SST	Simple Shoulder Test
WORC	Western Ontario Rotator Cuff Index
OSS	Oxford Shoulder Score
UCLA	University of California at Los Angeles Shoulder Rating Scale
DASH	Disabilities of the Arm, Shoulder, and Hand score
QuickDASH	Sports-specific module of the DASH score
ASORS	Athletic Shoulder Outcome Rating Scale
SF-12	12-Item Short Form Health Survey
PSF	Physical Component Summary
MSF	Mental Component Summary
PRISMA	Preferred Reporting Items for Systematic Reviews and Meta-Analyses
MINORS	Methodological Index for Nonrandomized Studies
LOE	Level of evidence
SSV	Subjective Shoulder Value
SSP	Supraspinatus
ISP	Infraspinatus
SSC	Subscapularis
TM	Teres minor
SLAP	Superior labrum from anterior to posterior tear
KJOC	Kerlan–Jobe Orthopaedic Clinic Shoulder and Elbow score
